# The Aggregation of Destabilized Ag Triangular Nanoplates and Its Application in Detection of Thiram Residues

**DOI:** 10.3390/nano12132152

**Published:** 2022-06-23

**Authors:** Chunhong Zhang, Hao Ren, Xiangkui Jiang, Guangfeng Jia, Zhigang Pan, Yongchun Liu

**Affiliations:** 1 Xi’an Key Laboratory of Advanced Control and Intelligent Process, School of Automation, Xi’an University of Posts & Telecommunications, Xi’an 710121, China; finespring2007@126.com (C.Z.); jiangxiangkui@xupt.edu.cn (X.J.); 2 Key Laboratory of Applied Surface and Colloid Chemistry, Ministry of Education, School of Chemistry and Chemical Engineering, Shaanxi Normal University, Xi’an 710062, China; renhao@snnu.edu.cn; 3 School of Electronic Information Engineering, Xi’an Technological University, Xi’an 710021, China; jgf97@126.com

**Keywords:** aggregation, Ag triangular nanoplates, LSPR, colorimetric detection, thiram

## Abstract

An aggregation or assembly of Ag triangular nanoplates (Ag TNPs) can cause dramatic changes in their optical properties, which is widely used in applications in the field of sensing. The assembly forms of nanoparticles are crucial for obtaining sensitive sensing signals, but it is unknown what kind of assembly dominates the aggregated Ag TNPs in aqueous solutions. Herein, using thiram-induced Ag TNP aggregation as a model, six different assembly models were established, including three planar (side-by-side, side-to-tip, and tip-to-tip) assemblies and three tridimensional (plane-to-plane, plane-to-tip, and plane-to-side) assemblies. The corresponding optical properties were then investigated. Both theoretical and experimental findings indicate that three-dimensional assemblies, especially plane-to-plane assembly, dominate the Ag TNPs aggregation solution, causing a blue shift of the absorption spectrum. Analysis of charge distribution patterns in Ag TNPs indicates that such a blue shift is caused by the electrostatic repulsive force in plane-to-plane assembly. Thus, we propose a simple colorimetric method for thiram detection using Ag TNPs as an indicator. The method exhibits a selective and sensitive response to thiram with a limit of detection of 0.13 μM in the range of 0.2–0.5 μM, as well as excellent performance in real samples like wheat.

## 1. Introduction

Thiram is a fungicide commonly used in the planting process. Due to its overuse, thiram residues cause great health problems, such as strong irritation to the skin and mucosa, and even liver damage [1,2]. Thus, it is very important to detect the presence of thiram residues in the environment. The traditional detection methods of thiram residues, including ion mobility spectrometry and high-pressure liquid chromatography, have to rely on expensive and complicated equipment [3,4]. Currently, some noble metal nanoparticles-based colorimetric detection methods of thiram residues have been developed. Chemically modified gold, silver, and copper nanoparticles have been applied to determine the concentration of thiram using their unique localized surface plasmon resonance (LSPR) effect [5,6,7,8,9,10,11]. Nevertheless, most of the methods fail to achieve a satisfactory limit of detection (LOD) or preparation procedures are too complex.

Nanoparticle aggregation-induced LSPR change is one of the most common nanoparticle-based sensing methods. Since nanoparticles in solution have a high surface energy, once the stabilizers that keep them dispersed are removed, they aggregate or assemble spontaneously. Different from other nanoparticles, Ag triangular nanoplates (TNPs) are notable for their sharp tips and anisotropic plate structures that produce more abundant signal variations [12,13,14,15,16]. Besides, because thiram’s disulfide bond is easily broken when exposed to the surface of Ag, it may act as an aggregation inducer of Ag TNPs by forming Ag-S bonds. As a result, the concentration of the thiram can be determined by observing the LSPR change of Ag TNPs.

On the other hand, different from other common morphologies, the aggregation/assembly morphology of TNPs is more complex because they have anisotropic morphologies, including three tips, three edges, and two planes. Therefore, there are at least six assembly modes in the aggregation process, including three kinds of planar (side-by-side, side-to-tip, and tip-to-tip) assembly forms and three kinds of tridimensional (plane-to-plane, plane-to-tip, and plane-to-side) assembly forms. Various assembly structures can result in different plasmonic coupling modes, so understanding the influence of various assemblies on LSPR is crucial for designing and optimizing sensors based on TNPs. Nevertheless, most studies focus on the planar assembly forms, such as bowtie nanostructure [17,18,19], coplanar nanoprism dimers with different rotation angles [20], edge-to-edge and plane-to-plane assemblies [21,22,23,24], but few have addressed three-dimensional assembly forms. Hence, it is still unclear how three-dimensional assembly forms affect Ag TNPs’ optical properties.

In this work, using thiram as an example, we examined the LSPR changes caused by the aggregation of Ag TNPs after replacing the stabilizers on the nanoplate surfaces with Ag-S bonds. Various assembly models were developed, and their spectra, local electric field (LEF), and charge distribution properties were calculated via the discrete dipole approximation (DDA). The physical mechanisms of the optical properties produced by various assembly structures were analyzed and clarified theoretically. A simple thiram residue detection method was proposed on the basis of changes in absorption spectra caused by thiram-induced Ag TNPs aggregation. Moreover, the selectivity of the method was confirmed, and its practicability was demonstrated using wheat samples.

## 2. Experimental Section

### 2.1. Chemicals and Materials

Pluronic F127 was purchased from Sigma-Aldrich (St. Louis, MO, USA). Sodium citrate (C_6_H_5_Na_3_O_7_∙2H_2_O) was purchased from Fuchen Chemical Reagents Factory (Tianjin, China). Silver nitrate (AgNO_3_), sodium borohydride (NaBH_4_), ascorbic acid (AA), and thiram were purchased from Aladdin. Acetonitrile was purchased from Fuyu Fine Chemical Co. Ltd. (Tianjin, China). Hydrogen peroxide (H_2_O_2_, 30 wt%) was purchased from Sinopharm Chemical Reagent Co. Ltd. (Shanghai, China). Metaldehyde, buprofezin, diethofencarb, chlorfenapyr, pymetrozine, and clofentezine were purchased from Hailier Chemical Company (Qingdao, China). Ethanol was purchased from Guanghua Sci-Tech Co. Ltd. (Shantou, China). All the chemicals used in our experiment are analytically pure. The ultrapure water used in our experiments was purified using a Millipore water purification system (Milli-Q, Millipore, Burlington, MA, USA) and had a resistivity of 18.2 MΩ cm.

### 2.2. Synthesis of Ag TNPs

The Ag TNPs were prepared using an Ag triangular nuclei-mediated growth method based on our previously reported works [25]. In a typical synthesis of seed solution, an aqueous solution of Pluronic F127 (2 g/L, 195 mL) was mixed with AgNO_3_ (50 mM, 400 μL), sodium citrate (75 mM, 4 mL), and H_2_O_2_ (30 wt%, 480 μL) at 20 °C. Then, an aqueous solution of NaBH_4_ (100 mM, 500 μL) was quickly injected into the mixture under vigorous stirring. After 15 min, the obtained triangular nuclei were centrifuged at 15,000 rpm for 15 min and redispersed in an aqueous solution of sodium citrate (1.5 mM, 200 mL) as the seed solution. To synthesize the Ag TNPs, AA (100 mM, 300 μL), 10 mL of acetonitrile, and 10 mL of the seed solution were added to 20 mL of ultrapure water at 0 °C. Under vigorous stirring, an aqueous solution of AgNO_3_ (50 mM, 150 μL) was dropwise injected into the above mixture at a rate of 18 μL/s. After 30 min, the Ag TNPs were obtained. The size of the Ag TNPs could be adjusted by changing the volume of AgNO_3_. Specifically, the volume of AgNO_3_ was 150, 220, and 300 μL for the synthesis of Ag TNPs with the main LSPR peak at 650, 700, and 750 nm, respectively. The ratio of the volume between AgNO_3_ and AA was 1:2.

### 2.3. Detection of Thiram Based on Ag TNPs

100 μL of thiram standard solution with different concentrations (0–7 μM) was added to 900 μL of Ag TNPs solution, respectively. The final concentrations of thiram were 0–0.7 μM, respectively. The mixture was stored at room temperature for 20 min and then the spectra of the reaction solution were recorded.

### 2.4. Detection of Thiram Residues in Wheat

A total of 1 mL of thiram standard solution with different concentrations (3, 3.5, and 4 μM) was added to 0.1 g of wheat samples. After drying, 1 mL of ethanol was added to the sample, followed by ultrasonic cleaning for 5 min, and the solution was collected. The final concentrations of thiram in the collected solutions were 0.3, 0.35, and 0.4 μM, respectively. A total of 100 μL of the collected solution was added to 900 μL of Ag TNPs solution. The mixture was stored at room temperature for 20 min and then the spectra of the reaction solution were recorded.

### 2.5. Characterization

The absorption spectra were recorded by means of a UV5 UV-Vis spectrophotometer (Mettler Toledo). A Tecnai G2 F20 (FEI) field transmission electron microscopy with an acceleration voltage of 75 kV was employed to acquire the transmission electron microscopy (TEM) images. The particle concentration of Ag TNPs used for deposition on the TEM grid is about 2.59 × 10^14^/L. The Litesizer 500 (Anto Paar, Graz, Austria) was employed to acquire the Zeta potentials. The cell used for Litesizer 500 (Anto Paar) to record the Zeta potentials is Omega Cuvette (Mat. No. 155765). The Raman spectra were analyzed with a Renishaw Raman inVia Reflex spectrometer (diode laser wavelength of 532 nm). The cryo-TEM image was recorded by a Titan Krios electron microscope (Thermo Fisher, Waltham, MA, USA) operating at 300 kV. A total of 3.5 μL of the detection solution were loaded onto the grid, and then the grid was blotted for 3.0 s and plunge-frozen in liquid ethane cooled by liquid nitrogen through a Vitrobot Mark IV (Thermo Fisher) at 4 °C and with 100% humidity. The grid was then measured.

### 2.6. Models and Methods of DDA

The models of Ag TNPs with different assembly structures have been constructed and the properties of spectra, LEF, and charge distributions have been calculated. In addition to the monomer Ag TNPs, the Ag TNPs were also assembled into structures of plane to plane, plane to tip, plane to side, side by side, side to tip, and tip to tip. In our calculations, the side length l of the Ag TNPs was fixed to 50 nm and the thickness h was fixed to 10 nm. The gap g between the two Ag TNPs was set as 1 nm, 5 nm, and 10 nm. The incident light with wavelength λ was oriented along the *x*-axis direction.

In this paper, a software package DDSCAT 7.3 which is based on the DDA algorithm was used to obtain the spectra, LEF, and charge distribution properties [26,27]. In these DDA theoretical calculations, we set the grid spacing of the cubic array as 1 nm. The monomer Ag TNPs with the least number of dipoles have more than 10,000 dipoles. According to the report of Sosa et al. [28], all the calculation results were reliable. In addition, the dielectric constants of silver used in our calculations were obtained from the report of Winsemius et al. [29] Water was chosen as the external dielectric environment.

## 3. Results and Discussion

### 3.1. Thiram-Initiated Aggregating of Ag TNPs

**Figure 1:**
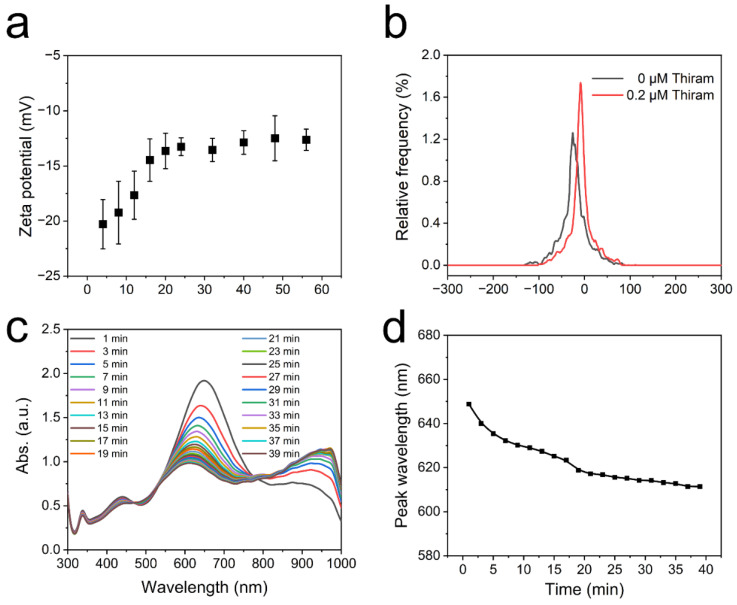
Zeta potential change trend with time by adding 0.2 μM of thiram (**a**). Zeta potentials of the Ag TNPs solution before and after adding thiram (**b**). The absorption spectra (**c**) and the peak wavelength change trend of the main absorption peak (**d**) with time by adding 0.3 μM of thiram into the Ag TNPs (the main absorption peak of the original Ag TNPs is located at 650 nm).

Thiram, a pesticide containing a disulfide bond, is capable of causing Ag TNPs to aggregate when it forms Ag-S bonds with the surface of the Ag TNPs (Figure S1) [30]. Ag-S bonds replace negatively charged citrate groups, changing the dispersion state and absorption spectra of Ag TNPs. In order to verify the interaction between Ag TNPs and thiram, the Zeta potentials of the reaction solution were recorded, as shown in Figure 1. With the addition of thiram (0.2 μM) to Ag TNPs, the negative surface charges decrease with time and reach a stable state after about 20 min (Figure 1a). Zeta potential now decreases to −13.3 mV from −31.4 mV (Figure 1b), showing that thiram has indeed replaced part of the negatively charged citrates and connected to the surface of the Ag TNPs.

After replacing the negatively charged citrate groups that stabilize the Ag TNPs, the surface charge will be reduced, thus promoting Ag TNPs aggregation. This process was clearly reflected by the time-dependent absorption spectra change. According to Figure 1c, the wavelength of the main absorption peak blueshifts and its value decreases with time. Moreover, the change in peak wavelength agrees with the trend of Zeta potential, and the rate of decrease slows until about 20 min (Figure 1d).

**Figure 2:**
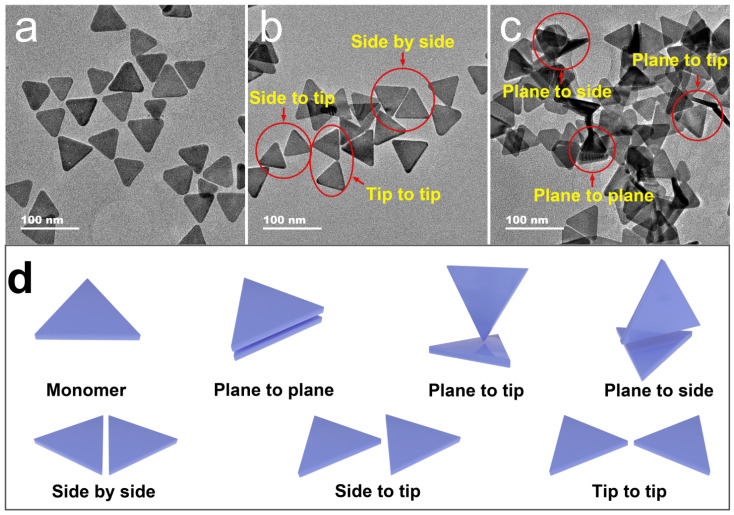
The TEM images of the Ag TNPs with the main absorption peak at 700 nm after adding different concentrations of thiram. The TEM images of the Ag TNPs with 0 μM (**a**), 0.4 μM (**b**), and 0.6 μM (**c**) of thiram. The schematic diagrams of different assembly forms of the stabilizer-removal induced Ag TNPs aggregates (**d**).

In order to observe the morphology of the Ag, TNPs aggregates under the action of thiram, different concentrations of thiram were added to the Ag TNPs solution and corresponding TEM images were taken (Figure 2a–c). As the dosage of thiram increases, we observe that the Ag TNPs aggregate more seriously. As shown in Figure 2a, the original Ag TNPs are uniformly dispersed. A certain amount of Ag TNPs present as planar assemblies (side-by-side, side-to-tip, and tip-to-tip assemblies) due to their interaction with the carbon film that covers the copper grid during drying. In the presence of a modest concentration of thiram (0.4 μM), Ag TNPs start to aggregate due to partial citrate groups being replaced, resulting in a small degree of plane-to-plane assembly (Figure 2b). Increasing thiram concentration to 0.6 μM causes Ag TNPs to aggregate very significantly. A large number of tridimensional assembly forms (plane-to-plane, plane-to-side, and plane-to-tip assemblies) appear (Figure 2c), which is dominated by plane-to-plane stacking because of the higher surface energy of the facets drives the information entropy [31]. According to these TEM images of aggregated Ag TNPs, the interaction between two Ag TNPs can be classified into six assembly forms, including plane-to-plane, plane-to-tip, plane-to-side, side-by-side, side-to-tip, and tip-to-tip assemblies (Figure 2d). These models were constructed and analyzed in the following theoretical calculations.

### 3.2. The Influence of Assembly Forms on LSPR Effect of the Ag TNPs

**Figure 3:**
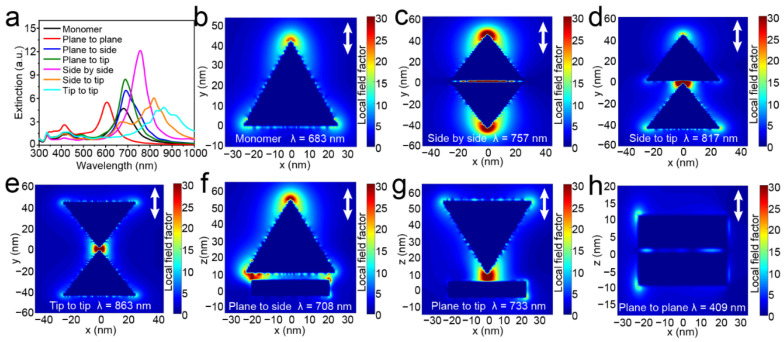
The extinction spectra of the monomer and the six assembly forms of Ag TNPs (the polarization was set to both along and perpendicular to the coupling direction) (**a**). The LEF distributions of the monomer (**b**) and the six assembly forms (**c**–**h**) of Ag TNPs (the polarization was set along the coupling direction, as shown by the arrow in the figures).

To reveal the principle of LSPR change of Ag TNPs in different assembly structures, the models of the aforementioned six assembly forms were established and their extinction spectra, LEF, and charge distributions were calculated by the DDA algorithm. As shown in Figure 3a, Figures S2 and S3, the extinction peaks of most of the assembled Ag TNPs show a redshift when compared with the monomer Ag TNP. This is consistent with the results of the most common spherical nanoparticles dimer [32]. In detail, the tip-to-tip form shows the biggest red shift followed by the side-to-tip, side-by-side, plane-to-tip, and plane-to-side forms. On the contrary, the extinction peak of the plane-to-plane structure has an obvious opposing blue shift. Combined with the TEM images of the Ag TNPs with thiram (Figure 2), the tridimensional assembly forms grow gradually with the increase of thiram concentration. Among them, the plane-to-plane assembly with a significant blue shift outweighs the two others with only a slight red shift. Therefore, the plane-to-plane assembly-induced blue shift is key to the design of LSPR sensors based on stabilizer removal induced aggregation of Ag TNPs.

Due to the resonance and coupling of the electrons on the surface of the nanoparticles, an extinction spectral change occurs, which is reflected in the LEF and charge distribution. Here, the polarization is set along the coupling direction of the nanoparticles to obtain their LEF properties. Figure 3b shows that for the monomer Ag TNP, the strong LEF enhancement regions are located at the sharp tips of the Ag TNPs. This is because the conduction electrons tend to concentrate at the sharp tips of the Ag TNPs. However, when two Ag TNPs approached one another, the plasmon coupling between them significantly altered the LEF. As the Ag TNPs aggregate into planar assemblies, the polarization direction was set along the *y*-axis to observe their interaction. The LEF enhancements are mostly located in the tips along the polarization direction and the gap between the two Ag TNPs (Figure 3c–e). As for the tridimensional assembly forms, the polarization direction was set along the *z*-axis. Based on the electron concentration function of the tip in the Ag TNPs and the electron resonance coupling between the two Ag TNPs, in the plane-to-tip form, a strong and concentrated LEF appears in the gap, while in the plane-to-side form, a LEF appears in the outer tip and the gap between tips and sides (Figure 3f,g). In the plane-to-plane form, the coupling direction is perpendicular to the plane. According to Figure 3h, the LEF enhancements were mostly distributed in the gap between the two planes and the outer tips of the Ag TNPs.

**Figure 4:**
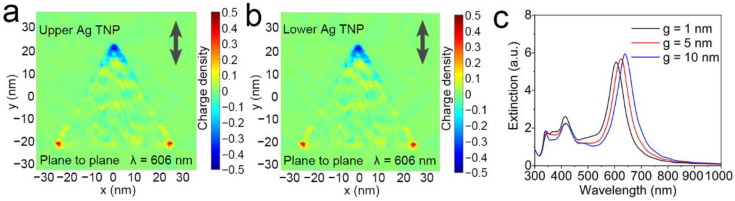
The charge distributions of the upper (**a**) and lower (**b**) Ag TNP in plane-to-plane form (the polarization was shown by the arrow in the figures). The extinction spectra of the plane-to-plane form of Ag TNPs with different gap values *g* (the polarization was set to both along and perpendicular to the coupling direction) (**c**).

Since plane-to-plane assembly of Ag TNPs exhibits similar spectral shifts to Ag TNPs aggregation induced by thiram, the charge distribution of plane-to-plane assembly was calculated and compared with other assembly forms. As illustrated in Figure 4a,b the positive and negative charges concentrated on the top and two bottom tips of the Ag TNPs, respectively, when polarization was along the plane of the plane-to-plane Ag TNPs. Both upper and lower Ag TNPs have the same charge distribution. So, the same charge distribution pattern produces electrostatic repulsive force, which increases the oscillation frequency between positive and negative charges and the SPR energy. Consequently, the peak wavelength blue-shifts. This principle has also been verified in plane-to-plane assemblies with different gaps. The main peak wavelength blue shifts as the gap decreases because of the increased electrostatic force as the distance between two Ag TNPs decreases (Figure 4c). On the other hand, for other assembly forms (Figure S4), they also have the same charge distribution pattern in the two Ag TNPs, but they have a strong reverse electric field in the gap between the two Ag TNPs. The electrostatic attraction force of the adjacent charge concentrated points in the gap lowers the vibration frequency of the positive and negative charges, resulting in a red shift in the peak wavelength.

### 3.3. Using the Aggregation of Ag TNPs for Thiram Detection

**Figure 5:**
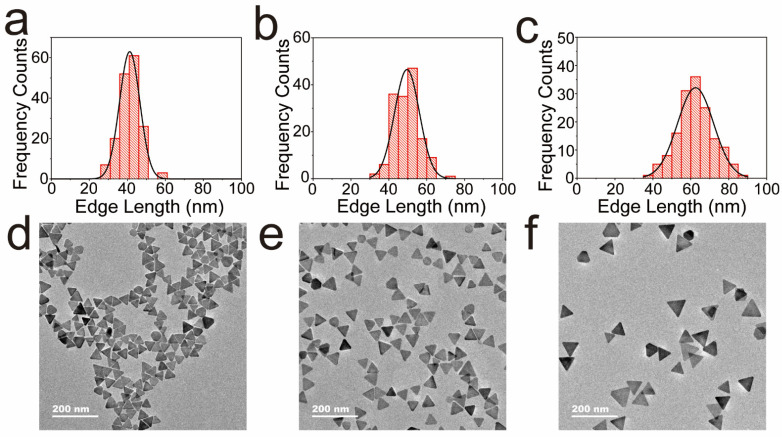
The histograms of the size distribution of the Ag TNPs with main absorption peaks at 650 nm (**a**), 700 nm (**b**), and 750 nm (**c**). The low magnification TEM images of the Ag TNPs with main absorption peaks at 650 nm (**d**), 700 nm (**e**), and 750 nm (**f**).

**Figure 6:**
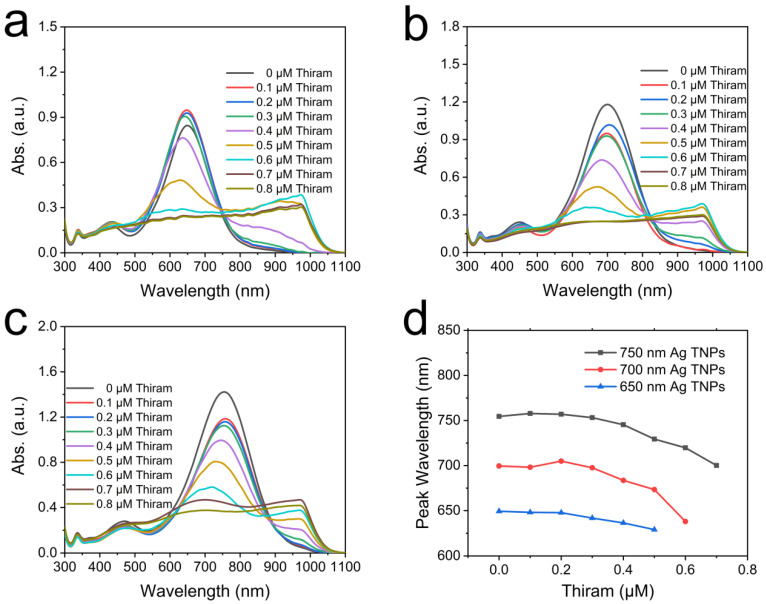
The absorption spectra of the Ag TNPs with main absorption peaks at 650 nm (**a**), 700 nm (**b**), and 750 nm (**c**) after adding different concentrations of thiram. The peak positions change the trend with the concentration of thiram increasing (**d**).

Thiram can be detected using the blue shift in the Ag TNPs spectrum. For the purpose of determining the optimal Ag TNPs size to detect thiram, we examined the impact of Ag TNPs sizes on the aggregation effect caused by thiram. Ag TNPs of various sizes were prepared using different AgNO_3_ dosages (the main absorption peak was centered at 650, 700, and 750 nm, respectively). Figure 5 shows a histogram of size distribution calculated from TEM images. The average edge lengths of Ag TNPs (main absorption peaks at 650 nm, 700 nm, and 750 nm) are 41 nm, 50 nm, and 63 nm, respectively. Various concentrations of thiram were then added to Ag TNPs of different sizes. As shown in Figure 6a–c, the spectra of Ag TNPs of different sizes blue-shift, and their values decrease as thiram concentration increases. Photographs of the reaction solution show a change in color from blue/green to gray, which indicates the aggregation of Ag TNPs and the possibility of colorimetric detection of thiram (Figure S5). The trend curves of absorption peak wavelengths of Ag TNPs in Figure 6d allow us to clearly determine which size has the best performance for thiram detection. For Ag TNPs of different sizes, the peak wavelength blue-shifts as the thiram concentration increases. Specifically, for Ag TNPs with main absorption peaks at 650 and 700 nm, the linearity range is 0.2–0.5 μM, the low value of which is smaller than for Ag TNPs with main absorption peaks at 750 nm (0.4–0.7 μM). Furthermore, compared with Ag TNPs with main absorption peaks at 650 nm, the peak wavelength blue shift of Ag TNPs with main absorption peaks at 700 nm is larger (31.6 nm). Therefore, Ag TNPs with the main absorption peak at 700 nm are the best candidates for the detection of thiram.

**Figure 7:**
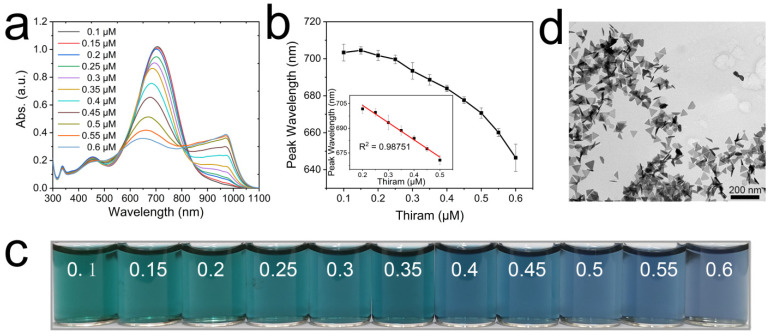
Ag TNPs-based detection of thiram. The absorption spectra of Ag TNPs by adding different concentrations of thiram (**a**). The curve of the main absorption peak shift with the concentration of thiram (**b**), the inset in (**b**) is the linear fitting results. The digital photograph corresponding to the above samples (**c**). The cryo-TEM image of the Ag TNPs with 0.6 μM of thiram (**d**).

Thiram was detected by using Ag TNPs with a main absorption peak at 700 nm. We measured the spectra of the Ag TNPs caused by adding different concentrations of thiram. Figure 7a shows that the main absorption peak gradually blue-shifts and the intensity decreases with increasing concentrations of thiram. As can also be seen in Figure 7b, the main peak wavelength is linearly related to the concentration of thiram. The linear range is 0.2–0.5 μM and the limit of detection (LOD) is 0.13 μM. Figure 7c shows a digital photograph of a solution of Ag TNPs containing different concentrations of thiram. The color of the solution gradually changed from green to dusty blue as the concentration of thiram increased. A statistically significant difference analysis of the experimental results has been conducted (Figure S6). A wavelength change is statistically significant when the concentration interval is 0.1 μM. Since the LOD for this method is 0.13 μM, the results are acceptable. The system also has the potential to detect other disulfide-containing molecules, such as tetraethylthiuram disulfide (Figure S7). To observe Ag TNPs aggregated in situ in the detection solution, cryo-TEM analysis was carried out. According to Figure 7d, we can see that there is a significantly higher number of tridimensional assemblies than that of planar assemblies in the detection solution. By combining the DDA calculated results, it can be concluded that the blue shifts in the spectra of thiram detection are mostly attributed to the tridimensional assemblies of Ag TNPs in particular plane-to-plane assembly.

**Figure 8:**
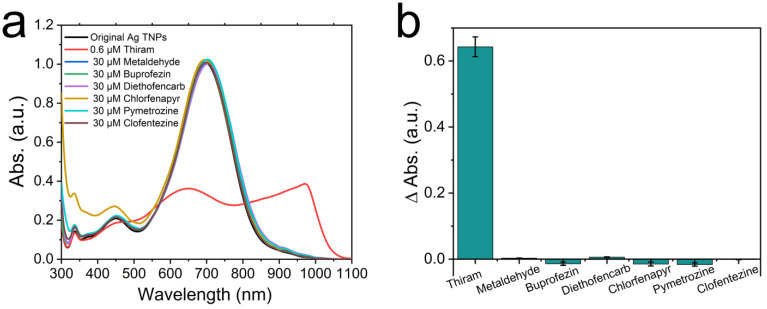
The selectivity of this method for detection of thiram. The absorption spectral characteristics (**a**) and the main absorption peak position changes (**b**) of the samples by adding thiram (0.6 μM) and other interfering pesticides (30 μM) relative to the sample without any pesticides, respectively.

To verify whether this method is selective for the detection of thiram, several commonly used pesticides were selected as interfering substances, where the concentration of thiram is 0.6 μM and that of the interfering pesticides is 30 μM. As shown in Figure 8 and Figure S8, the peak value and wavelength of the Ag TNPs with 0.6 μM of thiram changed evidently compared with the Ag TNPs without any pesticides, while those with the interfering pesticide whose concentration was 50 times greater than the thiram did not cause any spectral modification. The color of the Ag TNPs solution containing interfering pesticides is identical to that of the pesticide free solution. However, the sample containing thiram appears dusty blue due to its ability to aggregate. These results demonstrate that this method has a high selectivity for thiram detection.

Our Ag TNPs aggregation-based method performs well in real-world samples, such as thiram residues in wheat. We conducted spike-and-recovery experiments on wheat samples to verify the feasibility of this method. As listed in Table 1, the recoveries and the variation coefficients are in the range of 99.6–105% and 0.09–0.26%, respectively. Therefore, this method is highly reliable and has the potential to be applied to real-world samples to detect thiram residues. Furthermore, the performance of this method was compared to different colorimetric methods in the literature for the detection of thiram (Table S1). Among them, even though the ligand-free gold nanoparticles-based method has a better LOD and detection range, a preconcentration process by solid phase extraction is still required [6]. In our method, thiram can be detected without being altered or extracted. So, the detection method proposed in this manuscript is simpler.

## 4. Conclusions

In summary, we obtained the aggregated Ag TNPs with different planar and tridimensional assemblies by adding thiram to replace the citrate groups on the nanoparticle surfaces by forming Ag-S bonds, whose main peak wavelength blue-shifts as the thiram concentration increases. By comparing the spectral, LEF, and charge distribution characteristics of six different assembly models simulated by the DDA method, we found that only the extinction peak of plane-to-plane assembly showed obvious blue shift compared with the monomer, while other assembly forms showed red shift. TEM and spectra data indicate that plane-to-plane assembly dominates the Ag TNPs aggregation forms. Spectral blue shifts in Ag TNPs aggregation are caused by an increase in vibration frequency due to an electrostatic repulsive force generated between adjacent Ag TNPs with the same charge distribution patterns. In this way, a simple colorimetric detection of thiram was realized using stabilizer-removal-induced agglomeration of Ag TNPs without enteral modification. The linear range is 0.2–0.5 μM and the limit of detection (LOD) is 0.13 μM. The method shows excellent selectivity for thiram detection and excellent performance on real samples such as wheat. 

## Figures and Tables

**Table 1:** The performance of this method for detection of thiram residues in wheat.

Sample	Added (μM)	Found (μM)	Recovery (%)	RSD (%, *n* = 3)
Wheat	0.3	0.315	105	0.26
	0.35	0.348	99.6	0.20
	0.4	0.407	102	0.09

## Data Availability

The data presented in this study are available on request from the corresponding author.

## Supplementary Materials

The following supporting information can be downloaded at https://www.mdpi.com/article/10.3390/nano12132152/s1, Figure S1: The Raman properties of Ag TNP with 0.6 μM of thiram and the pure thiram. The main absorption peak of the Ag TNPs is at 700 nm; Figure S2: The extinction spectra of the monomer and the six assembly forms of Ag TNPs (the polarization was set along the coupling direction); Figure S3: The extinction spectra of the monomer and the six assembly forms of Ag TNPs (the polarization was set perpendicular to the coupling direction); Figure S4: The charge distributions of monomer, side-by-side, side-to-tip, tip-to-tip, plane-to-side, and plane-to-tip assembly form. The polarization was shown by the arrow in the figures; Figure S5: The digital photographs of the Ag TNPs with main absorption peaks at 650 nm (a), 700 nm (b), and 750 nm (c); Figure S6: The statistically significant differences analyses of the experimental results; Figure S7: The absorption spectra of the Ag TNPs with main absorption peak at 700 nm after adding different concentrations of tetraethylthiuram disulfide (a). The peak positions change trend with the concentration of tetraethylthiuram disulfide increasing (b); Figure S8: The digital photographs of the Ag TNPs after adding thiram (0.6 μM) and other interfering pesticides (30 μM) relative to the sample without any pesticides, respectively; Table S1: Performance comparison of different colorimetric methods for the detection of thiram. Reference [33] is cited in the supplementary materials.
