# Supplementary material for: The Aggregation of Destabilized Ag Triangular Nanoplates and Its Application in Detection of Thiram Residues
Source: Nanomaterials (Basel). 2022 Jun 23;12(13):2152. doi: 10.3390/nano12132152 (PMC9268207; doi:10.3390/nano12132152)
Supplement: Supplementary file 1 [file nanomaterials-12-02152-s001.zip › nanomaterials-1743603-supplementary.pdf]

Supplementary Materials

The Aggregation of Destabilized Ag Triangular Nanoplates and Its Application in Detection of Thiram Residues

Chunhong Zhang [1], Hao Ren [2], Xiangkui Jiang [1], Guangfeng Jia [3], Zhigang Pan [1,*] and Yongchun Liu [2,*]

[1] Xi'an Key Laboratory of Advanced Control and Intelligent Process, School of Automation, Xi'an University of Posts & Telecommunications, Xi'an 710121, China; finespring2007@126.com (C.Z.); jiangxiangkui@xupt.edu.cn (X.J.)
[2] Key Laboratory of Applied Surface and Colloid Chemistry, Ministry of Education, School of Chemistry and Chemical Engineering, Shaanxi Normal University, Xi'an 710062, China; renhao@snnu.edu.cn
[3] School of Electronic Information Engineering, Xi'an Technological University, Xi'an 710021, China; jgf97@126.com
* Correspondence: panzhigang0703@126.com (Z.P.); surfbioliu@snnu.edu.cn (Y.L.)

The Ag-S bond was characterized by SERS spectrum, as shown in Figure S1. One can clearly see the Ag-S mode at 233 cm^{-1}, which indicates the formation of Ag-S bond [1].

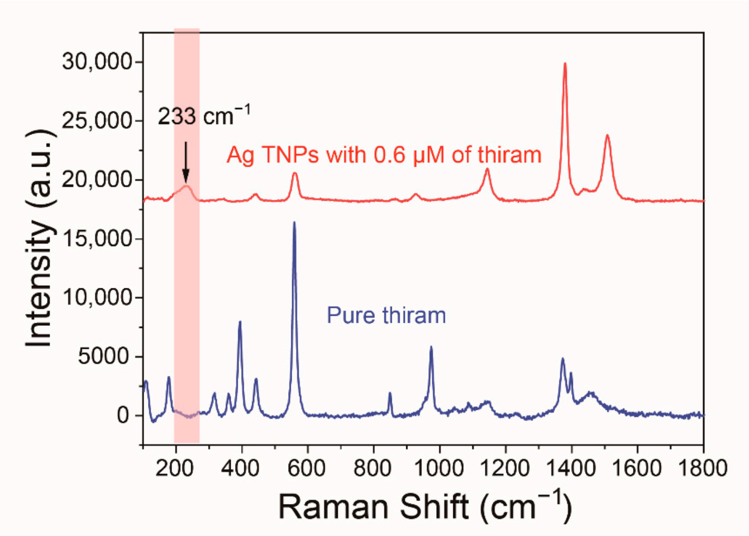

Figure S1. The Raman properties of Ag TNP with 0.6 μM of thiram and the pure thiram. The main absorption peak of the Ag TNPs is at 700 nm.

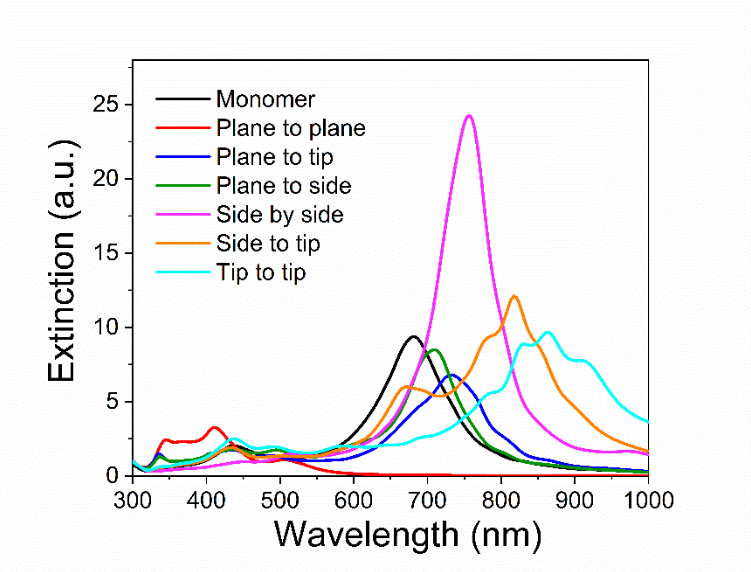

Figure S2. The extinction spectra of the monomer and the six assembly forms of Ag TNPs (the polarization was set along the coupling direction).

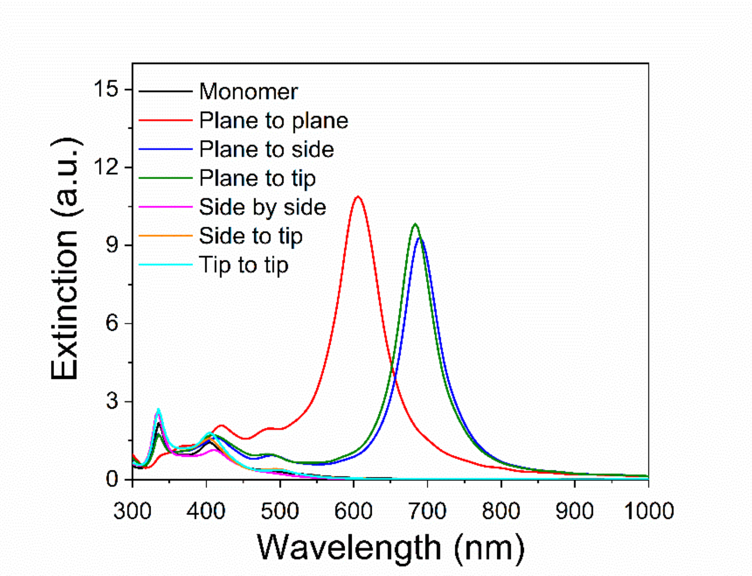

Figure S3. The extinction spectra of the monomer and the six assembly forms of Ag TNPs (the polarization was set perpendicular to the coupling direction).

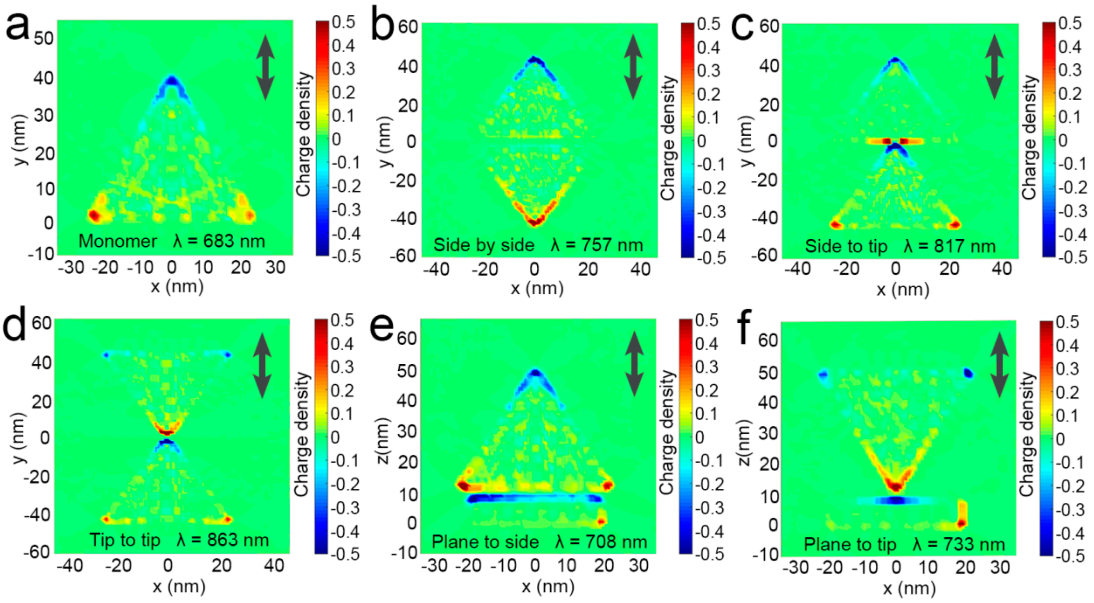

Figure S4. The charge distributions of monomer (**a**), side-by-side (**b**), side-to-tip (**c**), tip-to-tip (**d**), plane-to-side (**e**), and plane-to-tip (**f**) assembly form. The polarization was shown by the arrow in the figures.

All of the samples used for the deposition on the TEM grid are not concentrated or diluted. The Ag TNPs concentration is about 2.59 × 10^{14}/L.

The particle concentration of Ag TNPs was determined by argentometric titrations. In detail, 4 mL of Ag TNPs with the absorption peak at 700 nm was concentrated to 400 μL by centrifuging (10,000 rpm, 10 min). Nitric acid (100 μL, 10 mM) was added to the

concentrated Ag TNPs to dissolve the nanoparticle into silver ion. Then, potassium chromate (12.5 mM) was added until the solution became brick-red, and the volume of the used potassium chromate was recorded.

The particle concentration of Ag TNPs (C_{np}) can be calculated by blow formula:

$C_{Ag} = 2 * V_{pc} * C_{pc}/V_{np}$

$C_{np} = C_{Ag} * WM_{Ag}/(V_t * \varrho_{Ag})$

$Vt = 0.5 * L_{Ag} * H_{Ag} * T_{Ag}$

C_{Ag} is the concentration of Ag ions. C_{pc} is the concentration of potassium chromate (12.5 mM). V_{pc} is the volume of used potassium chromate (35 μL). V_{np} is the volume of Ag TNPs solution (4 mL). WM_{Ag} is the molecular weight of silver (107.8682). ϱ_{Ag} is the density of pure silver (10.5 g/mL). Vt is the average volume of single Ag TNP. L_{Ag} is the average edge length of Ag TNPs (50 nm). H_{Ag} is the average height of Ag TNPs. T_{Ag} is the average thickness of Ag TNPs (8 nm).

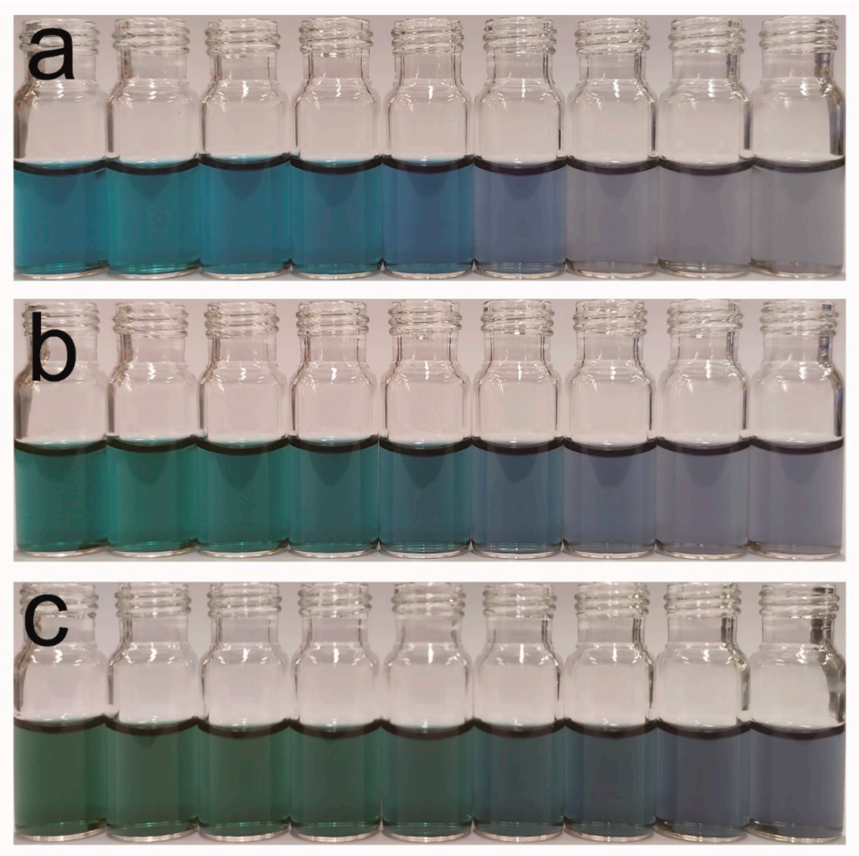

Figure S5. The digital photographs of the Ag TNPs with main absorption peaks at 650 nm (**a**), 700 nm (**b**), and 750 nm (**c**).

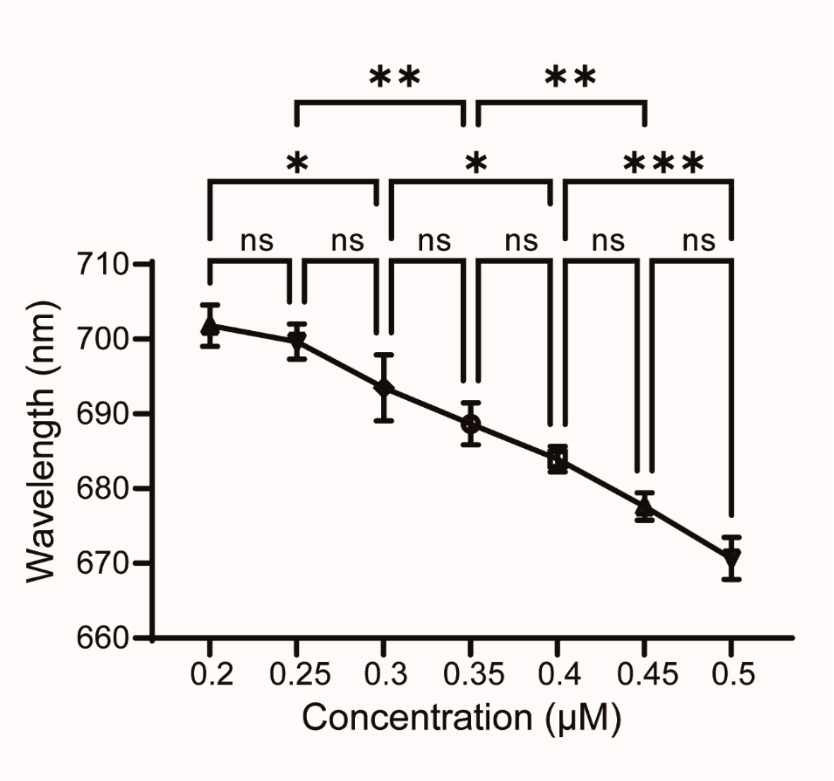

Figure S6. The statistically significant differences analyses of the experimental results. If a p-value is

bigger than 0.05, it is flagged with "ns". If a p-value is less than 0.05, it is flagged with one star (*). If a p-value is less than 0.01, it is flagged with 2 stars (**). If a p-value is less than 0.001, it is flagged with three stars (***).

Tetraethylthiuram disulfide, a commonly used drug for abstinence, was selected to be detected by the method proposed in this paper. The absorption spectra and the peak positions change trend are shown in Figure S7. Similar to thiram, the main absorption peak gradually blue shifted and the intensity decreased with the concentration of tetraethylthiuram disulfide increasing. These results can not only prove the aggregation mechanism proposed by us but also indicate the potential of our method for the detection of tetraethylthiuram disulfide.

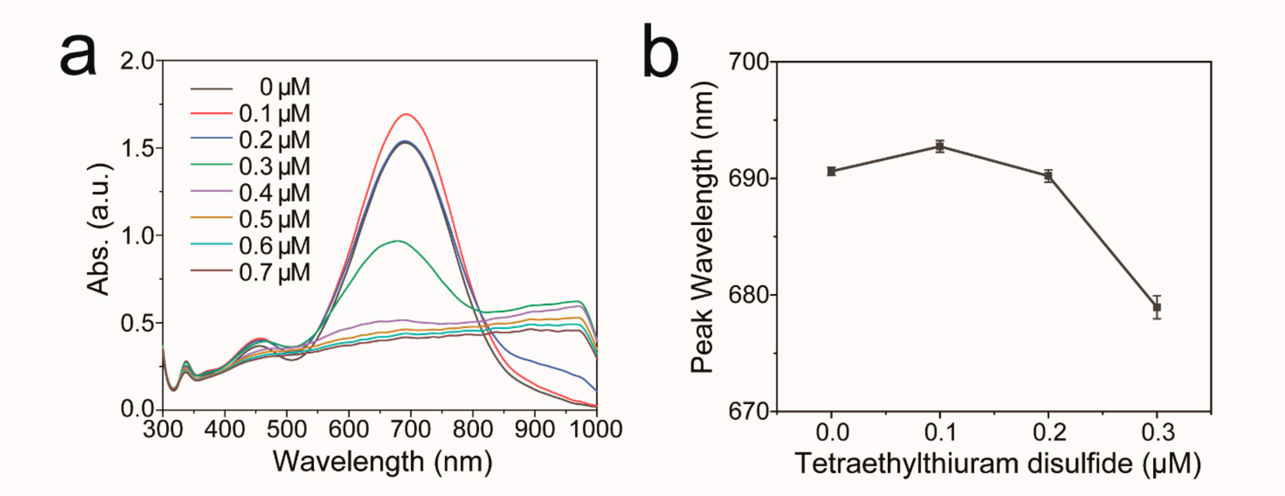

Figure S7. The absorption spectra of the Ag TNPs with main absorption peak at 700 nm after adding different concentrations of tetraethylthiuram disulfide (**a**). The peak positions change trend with the concentration of tetraethylthiuram disulfide increasing (**b**).

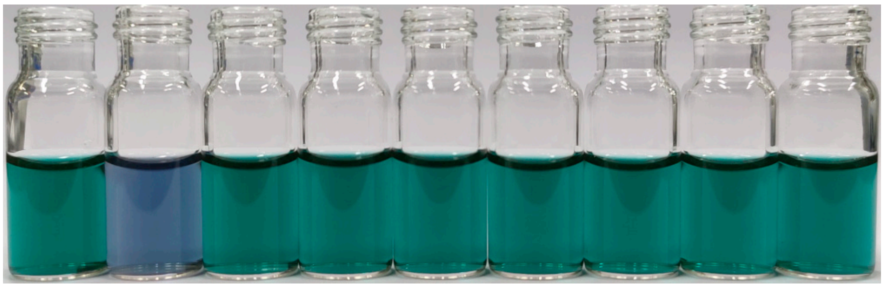

Figure S8. The digital photographs of the Ag TNPs after adding thiram (0.6 μM) and other interfering pesticides (30 μM) relative to the sample without any pesticides, respectively.

To evaluate the performance of this method, we compared it with different colorimetric methods for the detection of thiram in the literature, as shown in Table S1. Among them, although the reported method using ligand-free gold nanoparticles has a better LOD and detection range, a preconcentration process is necessary by solid phase extraction [3]. In our method, the detection of thiram can be realized without modification or extraction procedures. So, the detection method proposed in this manuscript is simpler.

Table S1. Performance comparison of different colorimetric methods for the detection of thiram.

Colorimetric methods	LOD	Detection linear range	Quantitative method	Extra modification
Gold nanoparticles [2]	0.17 μM	0.2–10 μM	Absorbance	No
Gold nanoparticles [3]	45 nM	0.1–0.73 μM	Absorbance ratio	No
Cyclen dithiocarbamate functionalized silver nanoparticles [4]	2.81 μM	10.0–20.0 μM	Absorbance ratio	Yes
Polyvinyl alcohol-decorated Au nanoparticles [5]	19.2 nM	0.02–8.32 μM	Reflectivity value ratio	Yes
Amine-functionalized Ag nanoparticles [6]	0.036 μM	0.1–100 μM	Absorbance ratio	Yes
Copper nanoparticles [7]	0.17 μM	0.5–25 μM	Absorbance ratio	No
Gold nanoparticles encoded with 4-aminothiophenol [8]	0.04 μM	0.05–2.0 μM	Absorbance ratio	Yes
**Triangular silver nanoplates (This work)**	**0.13 μM**	**0.2–0.5 μM**	**Absorbance**	**No**

References

1. Nyamekye, C.K.A.; Weibel, S.C.; Smith, E.A. Directional Raman scattering spectra of metal–sulfur bonds at smooth gold and silver substrates. *J. Raman Spectrosc.* **2021**, *52*, 1246–1255.
2. Rastegarzadeh, S.; Abdali, S. Colorimetric determination of thiram based on formation of gold nanoparticles using ascorbic acid. *Talanta* **2013**, *104*, 22–26.
3. Giannoulis, K.M.; Giokas, D.L.; Tsogas, G.Z.; Vlessidis, A.G. Ligand-free gold nanoparticles as colorimetric probes for the non-destructive determination of total dithiocarbamate pesticides after solid phase extraction. *Talanta* **2014**, *119*, 276–283.
4. Rohit, J.V.; Kailasa, S.K. Cyclen dithiocarbamate-functionalized silver nanoparticles as a probe for colorimetric sensing of thiram and paraquat pesticides via host–guest chemistry. *J. Nanopart. Res.* **2014**, *16*, 1–16.
5. Kong, L.; Huang, M.; Chen, J.; Lin, M. In situ detection of thiram in fruits and vegetables by colorimetry/surface-enhanced Raman spectroscopy. *Laser Phys.* **2020**, *30*, 065602.
6. Hoang, V.T.; Dinh, N.X.; Trang, N.L.N.; Khi, N.T.; Quy, N.V.; Tuan, P.A.; Tri, D.Q.; Thang, L.H.; Huy, T.Q.; Le, A.T. Functionalized silver nanoparticles-based efficient colorimetric platform: Effects of surface capping agents on the sensing response of thiram pesticide in environmental water samples. *Mater. Res. Bull.* **2021**, *139*, 111278
7. Anh, N.T.; Dinh, N.X.; Van, T.H.; Thuan, T.H.; Tung, L.M.; Le, V.P.; Tri, D.Q.; Le, A.T. Cost-effective tween 80-capped copper nanoparticles for ultrasensitive colorimetric detection of thiram pesticide in environmental water samples. *J. Nanomater.* **2021**, *2021*, 5513401.
8. Liu, K.; Jin, Y.; Wu, Y.; Liang, J. Simple and rapid colorimetric visualization of tetramethylthiuram disulfide (thiram) sensing based on anti-aggregation of gold nanoparticles. *Food Chem.* **2022**, *384*, 132223.
